# Pre- and perinatal risk factors for childhood leukaemia and other malignancies: a Scottish case control study

**DOI:** 10.1038/sj.bjc.6690609

**Published:** 1999-08

**Authors:** P A McKinney, E Juszczak, E Findlay, K Smith, C S Thomson

**Affiliations:** Scottish Cancer Intelligence Unit, ISD Scotland, South Trinity Road, Edinburgh, EH5 3SQ, UK

**Keywords:** childhood, leukaemia, cancer, epidemiology, risk factors, antenatal

## Abstract

A case control study of Scottish children aimed to identify risk factors for leukaemia and other cancers operating in the prenatal environment, during delivery and neonatally. Cases (0–14 years) were age-and sex- matched to two population-based controls and details abstracted from the mother's hospital obstetric notes. Analyses of 144 leukaemias (124 acute lymphoblastic leukaemias (ALL)), 45 lymphomas, 75 central nervous system (CNS) tumours and 126 ‘other solid tumours’ were conducted using conditional logistic regression. The presence of a neonatal infection significantly reduced the risk of ALL (odds ratio (OR) 0.49, 95% confidence interval (CI) 0.26–0.95), particularly in 0- to 4-year-olds. Positive swab tests confirmed 47% of ALL cases with any infection and 46% of controls. This is consistent with the hypothesis that early exposure to infections may reduce the risk of childhood ALL. Asphyxia at birth significantly increased the risk of leukaemia, which was accounted for by ALL. For the ‘other solid tumours’ higher levels of maternal education were inversely associated with risk (OR 0.59, 95% CI 0.37–0.94) but positively associated with antibiotics (OR 2.16 95% CI 1.10–4.25) and respiratory tract infections (OR 14.1, 95% CI 1.76–113.7) in pregnancy. No obvious plausible patterns of risk were detected either within or across disease subgroups. © 1999 Cancer Research Campaign


*British Journal of Cancer* (1999) **80**(11), 1844–1851
© 1999 Cancer Research Campaign
Article no. bjoc.1999.0609

### Pre- and perinatal risk factors for childhood leukaemia and other malignancies: a Scottish case control study

**PA McKinney, E Juszczak*, E Findlay, K Smith and CS Thomson**

Scottish Cancer Intelligence Unit, ISD Scotland, Trinity Park House, South Trinity Road, Edinburgh EH5 3SQ, UK;

**Summary** A case control study of Scottish children aimed to identify risk factors for leukaemia and other cancers operating in the prenatal environment, during delivery and neonatally. Cases (0–14 years) were age-and sex- matched to two population-based controls and details abstracted from the mother's hospital obstetric notes. Analyses of 144 leukaemias (124 acute lymphoblastic leukaemias (ALL)), 45 lymphomas, 75 central nervous system (CNS) tumours and 126 'other solid tumours' were conducted using conditional logistic regression. The presence of a neonatal infection significantly reduced the risk of ALL (odds ratio (OR) 0.49, 95% confidence interval (CI) 0.26–0.95), particularly in 0- to 4-year-olds. Positive swab tests confirmed 47% of ALL cases with any infection and 46% of controls. This is consistent with the hypothesis that early exposure to infections may reduce the risk of childhood ALL. Asphyxia at birth significantly increased the risk of leukaemia, which was accounted for by ALL. For the 'other solid tumours' higher levels of maternal education were inversely associated with risk (OR 0.59, 95% CI 0.37–0.94) but positively associated with antibiotics (OR 2.16 95% CI 1.10–4.25) and respiratory tract infections (OR 14.1, 95% CI 1.76–113.7) in pregnancy. No obvious plausible patterns of risk were detected either within or across disease subgroups.

**Keywords:** childhood; leukaemia; cancer; epidemiology; risk factors; antenatal

The considerable burden of childhood cancer, in terms of both morbidity and mortality, has generated extensive aetiological research over recent decades. Despite these efforts, apart from ionizing radiation, the causes of childhood leukaemia and other malignancies remain largely unexplained (Doll, 1989). The identification of risk factors has frequently focused on the vulnerable period of intrauterine growth and development, birth and neonatal life. The population-based Scottish case control study of childhood leukaemia and cancer has investigated potential risk factors occurring in the prenatal and perinatal period based on data abstracted from the hospital records of both mothers and neonates. A key aspect was to test prior hypotheses as well as identify associations for separate diagnostic subgroups and search for patterns of risk within and across distinct subsets of childhood malignancy.

### SUBJECTS AND METHODS

The cases studied were children (0–14 years) diagnosed with leukaemia or another malignancy during 1991–1994 while living in Scotland. The childhood population of Scotland at the 1991 census was 959 268. Pathological confirmation of diagnosis was required and cross-checks with the Scottish Cancer Registry and the UK National Register of Childhood Cancers (Stiller, 1995) optimized completeness of case ascertainment. Population-based controls matched on age (to within one calendar month), sex and health board area of residence were randomly selected from all eligible children registered for primary care. This sampling frame is considered appropriate representation of the general population in this age group (Roberts et al, 1995). An optimum two controls per case were selected and a full description of the study methodology is published elsewhere (McKinney et al, 1995). Scotland is a participating centre in the UK Children's Cancer Study (UKCCS).

The study was approved by the Local Research Ethics Committees of the 15 Health Boards in Scotland. Consultant clinicians or general practitioners gave consent to approach and interview mothers who gave their signed permission for information to be abstracted from their medical notes. In Scotland, 76 hospitals and maternity units gave access to medical records relating to births from 1976 to 1994. Records for births occurring in England and Wales (3.4%) were obtained by post.

Information from obstetric, delivery and neonatal records was recorded by two trained abstractors onto a specifically designed and highly structured standard form which has been validated in a previous study (Roman et al, 1997). Consistency and data quality were maximized through a programme of continual monitoring, coding checks and duplicate abstractions by the senior midwife abstractor (EF).

Demographic details of the mother were collected at interview. Each child was assigned a deprivation score (five categories representing quintiles of the Scottish population) according to Carstairs and Morris (1991) from the home address at the time of birth and its validated postcode. This was based on 1981 census data for pre-1986 births and the 1991 census thereafter. Illnesses were coded according to the 10th edition of the International Classification of Diseases (WHO, 1994) and the drug codes were classified using groups within the British National Formulary (British National Formulary, 1992).

The Birch and Marsden classification (Birch and Marsden, 1987) was used to define the following diagnostic subsets of cases – leukaemias, lymphomas, central nervous system (CNS) tumours and other solid tumours (neuroblastomas, retinoblastomas, renal,

*Received 17 September 1998*
*Revised 28 January 1999*
*Accepted 29 January 1999*

*Correspondence to*: PA McKinney

*Present address: Centre for Statistics in Medicine, Institute of Health Sciences, Old Road, Headington, Oxford OX3 7LF, UK.

**Table 1** Odds ratios (OR), 95% confidence intervals (CI) and numbers of exposed cases (ca) and controls (co) for demographic risk factors by diagnostic subgroup

| Mother | Category[a] | Total leukaemias OR (95% CI) Ca 144, Co 271 | Acute lymphoblastic leukaemia (subset) OR (95% CI) Ca 124, Co 236 | Lymphomas OR (95% CI) Ca 45, Co 82 | CNS tumours OR (95% CI) Ca 75, Co 133 | Other solid tumours OR (95% CI) Ca 126, Co 230 |
|---|---|---|---|---|---|---|
| School leaving age (years) | ≤ 16 vs > 16 | 0.77 (0.45–1.29) | 0.92 (0.53–1.60) | 1.40 (0.63–3.14) | 0.61 (0.30–1.25) | 0.75 (0.46–1.21) |
| | | 36, 77 | 33, 64 | 17, 25 | 14, 35 | 34, 72 |
| Educational qualifications | None vs some | 1.18 (0.73–1.91) | 1.22 (0.73–2.02) | 0.52 (0.22–1.26) | 0.73 (0.39–1.36) | 0.59[b] (0.37–0.94) |
| | | 100, 180 | 86, 155 | 27, 59 | 47, 92 | 73, 161 |
| Deprivation index at birth | I – least deprived | 1.00 | 1.00 | 1.00 | 1.00 | 1.00 |
| | | 24, 50 | 18, 45 | 8, 11 | 12, 17 | 16, 29 |
| | II | 0.92 (0.46–1.83) | 1.31 (0.62–2.76) | 1.01 (0.28–3.60) | 0.23 (0.06–0.86) | 0.89 (0.38–2.10) |
| | | 24, 53 | 23, 43 | 10, 13 | 7, 31 | 23, 40 |
| | III | 1.27 (0.64–2.48) | 1.38 (0.66–2.85) | 0.79 (0.21–3.00) | 0.96 (0.35–2.60) | 1.11 (0.47–2.67) |
| | | 30, 48 | 25, 44 | 9, 17 | 18, 23 | 23, 38 |
| | IV | 1.03 (0.53–2.03) | 1.32 (0.63–2.75) | 0.54 (0.13–2.25) | 0.79(0.26–2.38) | 0.86 (0.36–2.02) |
| | | 27, 53 | 24, 44 | 5, 14 | 13, 25 | 24, 46 |
| | V – most deprived | 1.18 (0.56–2.49) | 1.35 (0.60–3.03) | 0.53 (0.14–1.96) | 0.79 (0.28–2.24) | 0.77 (0.34–1.73) |
| | | 33, 60 | 28, 54 | 8, 17 | 18, 30 | 30, 63 |
| Ethnic group | White vs non-white | 1.00 (0.36–2.80) | 1.10 (0.38–3.16) | – | – | – |
| | | 6, 11 | 6, 10 | 2, 4 | 1, 1 | 3, 5 |

[a]Reference category stated first. [b]$P$ = 0.027.

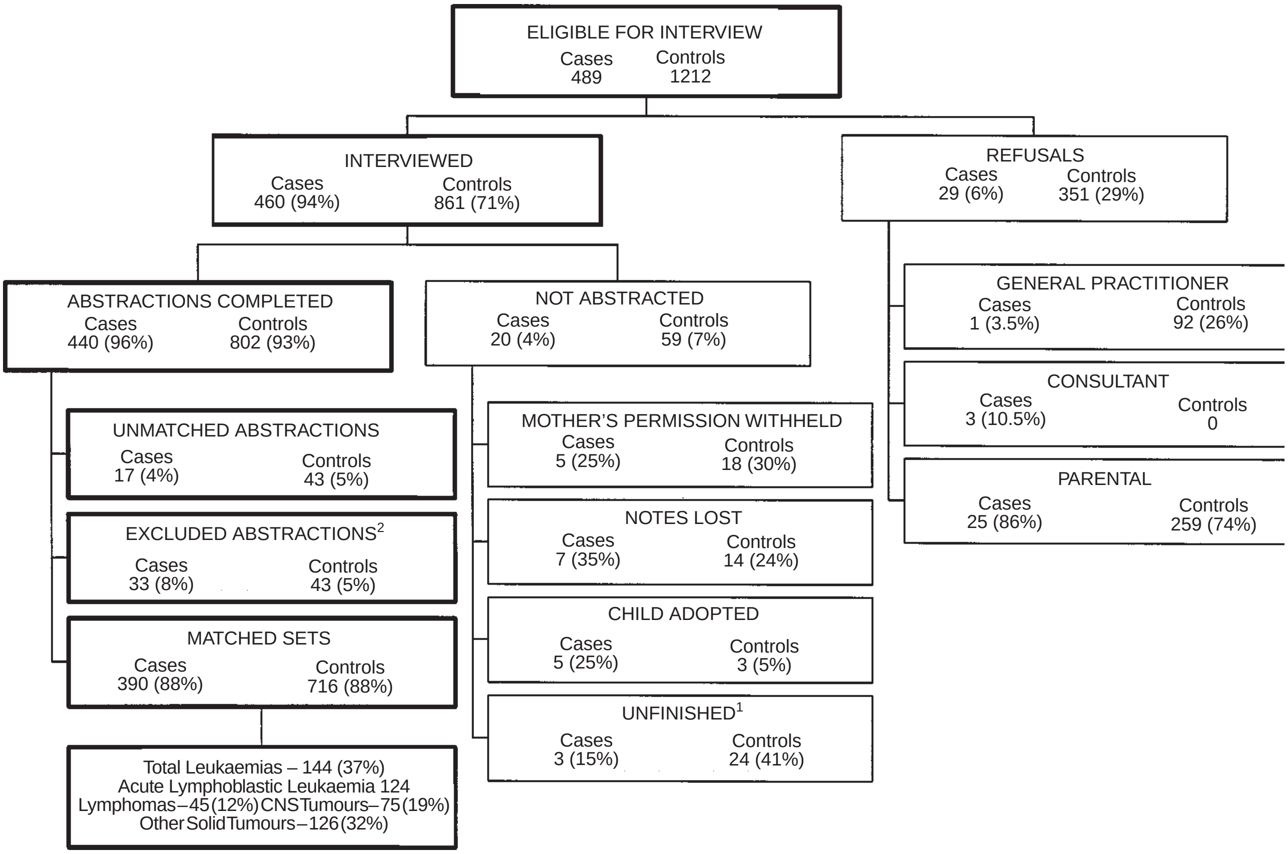

**Figure 1** Frequency of case and control interviews and obstetric abstractions. [1]Incomplete at end of study period. [2]Exclusions: Down's syndrome, multiple birth, diagnosed under 3 months hepatic, bone and germ cell tumours, soft tissue sarcomas and carcinomas) and a subset of the leukaemias, acute lymphoblastic leukaemia (ALL).

A profile of the study sample showing the original eligible cases and controls and the proportion of those who were interviewed, along with the proportion of interviewed subjects participating in

© 1999 Cancer Research Campaign

**Table 2** Odds ratios (OR), 95% confidence intervals (CI) and numbers of exposed cases (ca) and controls (co) for maternal pregnancy related risk factors by diagnostic subgroup

| Factor | Category[a] | Total leukaemias OR (95% CI) Ca 144, Co 271 | Acute lymphoblastic leukaemia (subset) OR (95% CI) Ca 124, Co 236 | Lymphomas OR (95% CI) Ca 45, Co 82 | CNS tumours OR (95% CI) Ca 75, Co 133 | Other solid tumours OR (95% CI) Ca 26, Co 230 |
|---|---|---|---|---|---|---|
| Mother's age at birth (years) | ≤19 | 1.86 (0.84–4.10) | 1.63 (0.69–3.85) | 3.14 (0.76–13.0) | 1.73 (0.49–6.10) | 0.85 (0.40–1.80) |
| | | 14, 15 | 11, 14 | 6, 5 | 6, 7 | 10, 23 |
| | 20–34 | 1.00 | 1.00 | 1.00 | 1.00 | 1.00 |
| | | 120, 234 | 104, 204 | 36, 75 | 65, 114 | 108, 185 |
| | ≥35 | 0.90 (0.41–1.95) | 0.98 (0.43–2.24) | 2.30 (0.38–14.1) | 0.60 (0.19–1.92) | 0.66 (0.28–1.55) |
| | | 10, 22 | 9, 18 | 3, 2 | 4, 12 | 8, 22 |
| Parity | 0 vs 1 or more | 0.82 (0.55–1.23) | 0.81 (0.52–1.25) | 1.11 (0.51–2.39) | 0.88 (0.50–1.55) | 1.19 (0.78–1.81) |
| | | 72, 148 | 61, 127 | 29, 51 | 40, 77 | 71, 119 |
| Gravidity | 1 vs more than 1 | 1.01 (0.67–1.51) | 0.95 (0.62–1.47) | 1.05 (0.49–2.27) | 1.20 (0.66–2.18) | 0.95 (0.61–1.48) |
| | | 87, 162 | 73, 140 | 29, 52 | 49, 84 | 77, 144 |
| Prior miscarriage | No vs yes | 1.28 (0.68–2.40) | 1.09 (0.54–2.17) | 0.26 (0.03–2.10) | 1.71 (0.66–4.44) | 0.51 (0.23–1.11) |
| | | 17, 26 | 13, 23 | 1, 7 | 8, 9 | 9, 29 |
| Infections: Any | None vs 1 or more | 1.43 (0.85–2.42) | 1.44 (0.81–2.55) | 0.82 (0.28–2.41) | 0.64 (0.30–1.34) | 1.81[b] (1.07–3.06) |
| | | 33, 46 | 26, 36 | 5, 12 | 13, 31 | 30, 32 |
| :Respiratory tract | None vs 1 or more | 1.46 (0.58–3.67) | 1.64 (0.60–4.46) | – | 0.38 (0.08–1.97) | 14.1[c] (1.76–113.7) |
| | | 9, 12 | 8, 10 | 1, 3 | 2, 9 | 8, 1 |
| :Viral | None vs 1 or more | – | – | – | – | – |
| | | 5, 8 | 4, 8 | 0, 1 | 0, 1 | 2, 3 |
| :Genitourinary | None vs 1 or more | 1.36 (0.66–2.83) | 1.18 (0.50–2.79) | 1.50 (0.34–6.70) | 0.91 (0.38–2.18) | 2.20 (0.95–5.08) |
| | | 13, 18 | 9, 14 | 3, 4 | 8, 14 | 13, 11 |
| :Fungal | None vs 1 or more | – | – | – | – | – |
| | | 2, 13 | 2, 11 | 1, 2 | 2, 4 | 5, 8 |
| Pre-eclampsia | No vs yes | 1.09 (0.66–1.82) | 1.19 (0.69–2.05) | 0.53 (0.19–1.51) | 0.77 (0.38–1.54) | 1.05 (0.61–1.82) |
| | | 34, 57 | 28, 45 | 6, 17 | 16, 34 | 26, 46 |
| Excessive vomiting | No vs yes | 0.49 (0.17–1.43) | 0.42 (0.13–1.35) | 0.64 (0.10–4.16) | 1.25 (0.46–3.43) | 0.92 (0.33–2.52) |
| | | 5, 17 | 4, 16 | 2, 5 | 7, 10 | 6, 12 |
| Ultrasound scans | None vs 1 or more | 1.07 (0.57–1.99) | 1.08 (0.56–2.09) | 0.32[d] (0.11–0.92) | 2.47 (0.86–7.08) | 2.01 (0.98–4.12) |
| | | 121, 228 | 104, 198 | 28, 65 | 69, 112 | 114, 192 |
| Abdominal X-rays | None vs 1 or more | 2.26 (0.69–7.45) | 2.50 (0.67–9.31) | 0.71 (0.17–2.97) | 1.11 (0.24–5.05) | 1.20 (0.29–5.02) |
| | | 6, 5 | 5, 4 | 3, 7 | 3, 4 | 3, 5 |
| Mode of delivery | Normal | 1.00 | 1.00 | 1.00 | 1.00 | 1.00 |
| | | 100, 98 | 84, 169 | 33, 59 | 59, 94 | 92, 163 |
| | Assisted | 1.05 (0.58–1.91) | 1.06 (0.56–2.00) | 0.89 (0.28–2.91) | 0.59 (0.24–1.45) | 0.61 (0.31–1.18) |
| | | 20, 38 | 18, 34 | 5, 11 | 8, 21 | 14, 41 |
| | Caesarean | 1.43 (0.79–2.59) | 1.43 (0.77–2.67) | 1.03 (0.36–3.01) | 0.68 (0.27–1.72) | 1.35 (0.71–2.57) |
| | | 24, 35 | 22, 33 | 7, 12 | 8, 18 | 20, 26 |

[a]Reference category stated first; [b]$P = 0.028$; [c]$P = 0.013$; [d]$P = 0.034$.

the obstetric data study, is shown in Figure 1. The other solid tumour group comprises 33 neuroblastomas, seven retinoblastomas, 23 renal, three hepatic, 13 bone and 23 germ cell tumours, 13 soft tissue sarcomas and 11 carcinomas and other cancers. Further details of the numbers of case and control mothers interviewed and response rates are published elsewhere (McKinney et al, 1998).

The statistical analysis excluded cases diagnosed under 3 months ($n = 16$), with Down's syndrome ($n = 5$) or from a multiple birth ($n = 12$) which resulted in the loss of 33 cases and their 43 matched controls. In addition, as the analysis was restricted to matched sets, information was lost for a further 17 cases (4%) lacking controls and 43 controls (5%) without a matched case. These were distributed equally across the diagnosis groups. A standard case control analysis (Breslow and Day, 1980) of 390 matched case control sets (326 triplets, 64 pairs) was performed using Stata (StataCorp, 1997). Odds ratios (OR), 95% confidence intervals (CI) and $P$-values were estimated using conditional logistic regression. The presentation of ORs is restricted to when the prevalence of an exposure was at least 5% in either cases or controls. When appropriate and unless otherwise stated, missing values were included in the reference category.

### RESULTS

Table 1 shows results for demographic factors. There was a reduction in the risk of CNS tumours and other solid tumours, the latter reaching statistical significance, both with increased levels of education and school-leaving age; the children of mothers who were more highly educated were at lower risk. There was no evidence of any trend in risk of any of the malignancies studied in relation to levels of deprivation.

Results for pregnancy-related factors are shown in Table 2. The only statistically significant risks were a positive association with infections during pregnancy for the other solid tumours and an OR < 1 for the lymphomas with ultrasound examinations. The infections were accounted for by respiratory tract infections, of which the majority (5/8 cases) occurred in the third trimester. There was no association of risk with increasing age of mother in any subgroup. Drugs ingested during pregnancy (Table 3) reflected the

 © 1999 Cancer Research Campaign

**Table 3** Odds ratios (OR), 95% confidence intervals (CI) and numbers of exposed cases (ca) and controls (co) for drugs taken in pregnancy

| | Total leukaemias OR (95% CI) (Ca 144, Co 271) | Acute lymphoblastic leukaemia (subset) OR (95% CI) (Ca 124, Co 236) | Lymphomas OR (95% CI) (Ca 45, Co 82) | CNS tumours OR (95% CI) (Ca 75, Co 133) | Other solid tumours OR (95% CI) (Ca 126, Co 230) |
|---|---|---|---|---|---|
| Antacids | 1.15 (0.52–2.55) | 1.24 (0.50–3.06) | 1.11 (0.24–5.20) | 1.27 (0.39–4.14) | 0.97 (0.42–2.22) |
| | 11, 17 | 9, 13 | 3, 5 | 6, 9 | 9, 17 |
| Hypnotics and anxiolytics | 1.45 (0.67–3.14) | 2.04 (0.88–4.74) | 0.43 (0.11–1.63) | 1.36 (0.57–3.24) | 0.88 (0.41–1.90) |
| | 13, 17 | 13, 13 | 3, 12 | 9, 12 | 11, 23 |
| Analgesics | 1.33 (0.68–2.62) | 1.30 (0.60–2.82) | 0.58 (0.14–2.35) | 0.86 (0.29–2.55) | 0.97 (0.45–2.11) |
| | 15, 21 | 11, 16 | 3, 9 | 5, 10 | 12, 24 |
| Antibiotics | 1.60 (0.92–2.80) | 1.74 (0.95–3.20) | 0.75 (0.20–2.83) | 0.65 (0.28–1.46) | 2.16[b] (1.10–4.25) |
| | 26, 34 | 23, 28 | 3, 8 | 9, 23 | 21, 19 |
| Topical anti-fungals | 0.10[a] (0.01–0.75) | 0.14 (0.02–1.09) | 1.00 (0.25–4.00) | 0.74 (0.24–2.23) | 1.74 (0.82–3.68) |
| | 1, 18 | 1, 14 | 3, 6 | 5, 12 | 14, 15 |
| Iron preparations | 0.95 (0.62–1.45) | 0.80 (0.51–1.25) | 0.79 (0.36–1.73) | 0.89 (0.51–1.58) | 1.03 (0.66–1.60) |
| | 75, 143 | 57, 120 | 20, 41 | 39, 75 | 70, 129 |
| Anti-nauseants | – | – | 1.88 (0.39–8.92) | – | 2.02 (0.67–6.12) |
| | 6, 4 | 4, 2 | 4, 5 | 2, 1 | 7, 7 |
| General anaesthetics | – | – | – | – | – |
| | 4, 2 | 4, 2 | 0, 1 | 3, 0 | 2, 0 |

[a]$P = 0.025$; [b]$P = 0.026$.

observed increase in the risk of infections for the solid tumour group with a raised risk for antibiotics. An inverse association was observed for use of topical antifungals in the leukaemias and ALL groups, although the OR was not statistically significant for the ALLs. Adjustment for maternal age, which is potentially associated with increased attendance at hospital, showed no evidence of confounding this result.

For drugs taken in labour (not tabulated), which included analgesia, there were no significantly positive or negative ORs with the exception of a reduced risk for antacid use for the CNS tumours (cases 14, controls 39, OR 0.33, 95% CI 0.13–0.89).

Neonatal risk factors are presented in Table 4. A significantly reduced risk for neonatal infections is observed for ALL, accounted for by 'skin infections' which mainly comprise omphalitis or infection of the umbilical cord. For ALL, 15 (12.1%) cases and 52 (22.0%) controls were recorded with 'any infection' and two (1.6%) cases and 19 (8.1%) controls with 'skin infections'. For 'any infection' in the ALLs, 7/15 (47%) cases and 24/52 (46%) were confirmed by positive swab test results. Separate adjustment of the significant OR for 'any infection' and skin infections by the other significant associated variables, Apgar scores and birth asphyxiation had no effect on the magnitude or significance of the OR. Adjusting by deprivation score (five categories) and maternal school leaving age (< 16 vs 16 or older), on the grounds that the occurrence of leukaemia may be influenced by social class, had little effect on the significance or magnitude of the OR. The appearance of neonatal infections may be related to mode of delivery with fewer infections expected for caesarean deliveries. Adjustment for mode of delivery did not affect any neonatal infections results for the leukaemia group. The risk of skin infections is significantly raised for the 'other solid tumours' with 6/14 cases being diagnosed with Wilm's tumour.

Two linked exposures, poor Apgar scores (4–6) at 1 min, and birth asphyxia (ICD-10 P21), had significantly raised OR for the leukaemias and ALLs, except for Apgars 4–6 for the ALLs which only approached significance (see Table 4). Proportions of cases and controls exposed for leukaemias were: Apgars 4–6 cases 18.8%, controls 11.1%; birth asphyxia cases 11.1%, controls 4.8%; and correspondingly for ALL cases 19.4%, controls 11.4% and cases 10.5%, controls 4.7% respectively. At 5 min, no children had an Apgar score of 3 or less and for 4–6 the OR was close to unity and non-significant.

Table 5 shows details of infectious exposure in the leukaemias. For all infections taken together, the protective effect is seen to be significant in the 0 to 4-year-old ALLs, but not in those aged 5–14 years. The high proportion of exposed controls in the 0 to 4-years-olds (25.3%) compared to the 5–14 group (16.3%) was reversed for the CNS tumours (0–4, 14.0%; 5–14, 19.3%), reduced for the 'other solid tumours' (0–4, 21.1%; 5–14, 15.9%) and similar for the smaller group of lymphomas (0–4, 23.8%; 5–14, 16.4%).

### DISCUSSION

A key strength of the population-based Scottish case control study was the use of medical records as the principal source of information thus avoiding reliance on self-reporting of illness, which is particularly susceptible to recall bias. A very high proportion of notes were abstracted both for case and control mothers, despite elapsed times of up to 20 years between birth and data collection.

Data abstraction was restricted to two trained researchers, one of whom was a midwife. Although not blind to case control status, the abstractors had no knowledge of the case diagnosis and were unaware of the hypotheses under investigation. The use of a specifically designed highly structured and previously validated data collection form (Roman et al, 1997) reduced the likelihood of systematic bias. The method of analysis was conservative, using only matched sets, resulting in the loss of a small amount of data, but this was proportionately distributed between the cases and controls and across diagnostic groups.

The entire study was designed to collect interview data along with further independent sets of information from obstetric and general practitioner records. Recruitment into the interview phase of the study may have been subject to bias as the refusal rate for eligible cases was 6% compared to 26% for controls. However,

**Table 4** Odds ratios (OR), 95% confidence intervals (CI) and numbers of exposed cases (ca) and controls (co) for neonatal risk factors by diagnostic subgroup

| Factor | Category[a] | Total leukaemias OR (95% CI) Ca 144, Co 271 | Acute lymphoblastic leukaemia (subset) OR (95% CI) Ca 124, Co 236 | Lymphomas OR (95% CI) Ca 45, Co 82 | CNS tumours OR (95% CI) Ca 75, Co 133 | Other solid tumours OR (95% CI) Ca 126, Co 230 |
|---|---|---|---|---|---|---|
| Gestation (weeks) | 38–42 vs < 38 | 1.00 (0.49–2.03) | 0.93 (0.44–1.97) | 0.51 (0.13–1.93) | 1.00 (0.42–2.40) | 0.93 (0.43–2.01) |
| | | 14, 26 | 12, 24 | 4, 12 | 9, 16 | 12, 24 |
| Birthweight (g) | < 2500 | 1.84 (0.67–5.04) | 1.52 (0.52–4.43) | 2.03 (0.42–9.75) | 1.36 (0.35–5.26) | – |
| | | 9, 10 | 7, 10 | 4, 4 | 4, 5 | 5, 11 |
| | 2500–3499 | 1.00 | 1.00 | 1.00 | 1.00 | 1.00 |
| | | 74, 149 | 64, 131 | 21, 40 | 39, 69 | 70, 119 |
| | ≥ 3500 | 1.12 (0.73–1.73) | 1.17 (0.74–1.85) | 0.99 (0.43–2.28) | 0.99 (0.54–1.83) | 0.84 (0.54–1.33) |
| | | 60, 112 | 52, 95 | 19, 38 | 32, 58 | 51, 100 |
| Admitted to special care | No vs yes | 1.53 (0.93–2.50) | 1.56 (0.93–2.62) | 0.70 (0.29–1.69) | 0.85 (0.38–1.89) | 1.07 (0.61–1.90) |
| Unit | | 40, 58 | 36, 52 | 10, 22 | 12, 24 | 28, 49 |
| Jaundice | No vs yes | 0.79 (0.51–1.20) | 0.77 (0.49–1.21) | 0.67 (0.32–1.44) | 0.87 (0.48–1.58) | 1.04 (0.66–1.63) |
| | | 49, 109 | 44, 100 | 20, 44 | 30, 58 | 46, 82 |
| Initially breastfed | No vs yes | 0.96 (0.62–1.49) | 0.92 (0.58–1.47) | 0.86 (0.41–1.84) | 1.39 (0.72–2.67) | 0.73 (0.44–1.19) |
| | | 52, 99 | 44, 87 | 18, 36 | 31, 47 | 38, 82 |
| Neonatal Infections | No vs yes | 0.62 (0.34–1.11) | 0.49[d] (0.26–0.95) | 1.18 (0.50–2.80) | 0.91 (0.38–2.15) | 0.97 (0.55–1.71) |
| Any | | 19, 55 | 15, 52 | 10, 15 | 12, 23 | 22, 43 |
| Conjunctivitis | No vs yes | 0.76 (0.38–1.51) | 0.63 (0.29–1.36) | 1.43 (0.55–3.68) | 0.81 (0.30–2.21) | 0.56 (0.27–1.16) |
| | | 13, 33 | 10, 30 | 9, 11 | 7, 15 | 11, 34 |
| Skin | No vs yes | 0.20[b] (0.05–0.87) | 0.20[e] (0.05–0.87) | – | 0.40 (0.08–2.10) | 3.24[f] (1.35–7.78) |
| | | 2, 19 | 2, 19 | 2, 4 | 2, 8 | 14, 9 |
| Apgar at 1 min | 7+ | 1.00 | 1.00 | 1.00 | 1.00 | 1.00 |
| | | 111, 225 | 97, 195 | 38, 67 | 65, 109 | 106, 185 |
| | 4–6 | 1.81[c] (1.03–3.19) | 1.79 (0.99–3.24) | 0.63 (0.19–2.09) | 0.66 (0.22–1.96) | 0.66 (0.30–1.44) |
| | | 27, 30 | 24, 27 | 4, 10 | 6, 15 | 10, 27 |
| | 1–3 | – | – | – | – | – |
| | | 4, 8 | 2, 7 | 0, 3 | 3, 4 | 5, 9 |
| Problems at birth | | | | | | |
| Complications of cord | No vs yes | 1.36 (0.69–2.68) | 1.10 (0.52–2.33) | 1.84 (0.46–7.41) | 1.21 (0.43–3.44) | 0.61 (0.29–1.26) |
| | | 17, 23 | 13, 22 | 4, 4 | 7, 10 | 11, 31 |
| Cyanosis | No vs yes | 0.57 (0.26–1.24) | 0.53 (0.23–1.22) | 1.90 (0.57–6.39) | 0.48 (0.15–1.51) | 0.87 (0.41–1.83) |
| | | 9, 28 | 8, 26 | 6, 6 | 4, 15 | 11, 23 |
| Vomiting | No vs yes | 0.63 (0.22–1.77) | 0.63 (0.20–1.97) | 1.56 (0.24–10.1) | 1.08 (0.28–4.18) | 1.36 (0.57–3.26) |
| | | 5, 15 | 4, 12 | 3, 4 | 5, 7 | 9, 13 |
| Cephalohaematoma | No vs yes | 0.88 (0.34–2.33) | 0.77 (0.26–2.34) | 1.79 (0.36–8.97) | 1.15 (0.32–4.14) | – |
| scalp injury | | 7, 15 | 5, 12 | 3, 3 | 4, 6 | 1, 8 |
| Birth asphyxiation | No vs yes | 2.44[g] (1.15–5.20) | 2.41[h] (1.05–5.52) | 1.79 (0.36–8.97) | – | 1.21(0.52–2.80) |
| | | 16, 13 | 13, 11 | 3, 3 | 3, 4 | 10, 15 |

[a]Reference category stated first. [b]$P = 0.032$; [c]$P = 0.038$; [d]$P = 0.034$; [e]$P = 0.032$; [f]$P = 0.008$; [g]$P = 0.020$; [h]$P = 0.039$.

66% of the controls were the 'first choice' mothers to be interviewed and the geographical pattern and age distribution of the refusers did not differ between cases and controls.

The testing of case control differences in over 200 variables, resulted in 13 ORs significantly greater or less than unity. Multiple testing and small numbers mean these results could potentially be explained by chance. However, interpretation of their significance will depend on the existence of a prior hypothesis, biological plausibility, the presence of a 'dose–response' and consistency with other published reports of the same effect. The results from the present study are described by subgroup and discussed in the context of findings from other studies. A study of neonatal exposure to Vitamin K is already published (McKinney et al, 1998).

### Leukaemias and acute lymphoblastic leukaemia

One striking finding was the significant deficit of neonatal infections for children who later developed ALL, applying especially to those diagnosed between the ages of 0 and 4 years. Protection appears to be most prominent for children diagnosed in the 'childhood peak' of ALL, which mainly comprises the common pre-B-cell immunophenotype. The negative association for neonatal infections supports the hypothesis proposed by Greaves and Alexander (1993). This postulates that a lack of exposure to infections in infancy and immunological isolation increases the risk of common ALL, because in the absence of early exposure the immune system may remain 'unprogrammed' and unable to respond appropriately later in life (Greaves, 1997).

The restriction of the protective effect of infections to the young leukaemias is unlikely to be artefactual. The differential frequency of infections by age group in the ALLs is clearly not present in the CNS group, although it is seen to a lesser extent in the small lymphoma group and the heterogeneous 'other solid tumours'.

A significant excess of control children had 'any infection', mainly accounted for by skin infections. Clinical observations of an infection were accompanied by confirmed positive laboratory swab tests for approximately half of all the infections for both cases and controls.

To address the issue of confounding, adjustment was made for the other significant variables and those considered to an appro-

 *© 1999 Cancer Research Campaign*

**Table 5** Frequencies, odds ratios (OR) and 95% confidence intervals (CI) of neonatal infections for the leukaemias and acute lymphoblastic leukaemias by age

| | | Total leukaemias ($n$ = 144) | | | Acute lymphoblastic leukaemias ($n$ = 124) | | |
|---|---|---|---|---|---|---|---|
| **Infection** | **Age group** | **Cases $n$ (%)** | **Controls $n$ (%)** | **OR (95% CI)** | **Cases $n$ (%)** | **Controls $n$ (%)** | **OR (95% CI)** |
| Any (1 or more) | 0–4 | 10 (11.8) | 39 (23.6) | 0.40[a] (0.17–0.90) | 9 (11.7) | 38 (25.3) | 0.35[b] (0.15–0.83) |
| | 5–14 | 9 (15.3) | 16 (15.1) | 1.10 (0.47–2.59) | 6 (12.8) | 14 (16.3) | 0.85 (0.32–2.27) |
| Respiratory tract | 0–4 | 0 | 1 | – | 0 | 1 | – |
| | 5–14 | 0 | 1 | – | 0 | 1 | – |
| Gastrointestinal tract | 0–4 | 0 | 0 | – | 0 | 0 | – |
| | 5–14 | 0 | 1 | – | 0 | 1 | – |
| Fungal | 0–4 | 1 | 3 | – | 1 | 3 | – |
| | 5–14 | 1 | 0 | – | 0 | 0 | – |
| Skin | 0–4 | 2 (2.4) | 14 (8.5) | 0.27 (0.06–1.21) | 2 (2.6) | 14 (9.3) | 0.27 (0.06–1.21) |
| | 5–14 | 0 | 5 | – | 0 | 5 | – |
| Conjunctivitis | 0–4 | 7 (8.2) | 23 (13.9) | 0.55 (0.22–1.40) | 6 (7.8) | 22 (14.7) | 0.47 (0.17–1.30) |
| | 5–14 | 6 (10.2) | 10 (9.4) | 1.22 (0.43–3.47) | 4 (8.5) | 8 (9.3) | 1.00 (0.30–3.32) |
| Other[c] | 0–4 | 1 | 3 | – | 1 | 3 | – |
| | 5–14 | 3 | 1 | – | 2 | 1 | – |

[a]$P$ = 0.027; [b]$P$ = 0.017; [c]Includes ICD-10 P36 Bacterial sepsis of newborn and P39.9 Infection specific to the perinatal period, unspecified.

priate proxy for social class, i.e. deprivation and maternal school-leaving age. The results remained unaffected and therefore unexplained by social class, which is perhaps not surprising as neither deprivation nor school-leaving age are significant in the univariate analysis. If lower social class is related to higher infectious exposure the general direction of the results are not supportive of the infections hypothesis. Caesarean delivery is likely to result in reduced exposure to infection compared to vaginal delivery, but the neonatal infections remained independent of this potential confounder.

A negative association was observed for maternal use of topical antifungals, primarily pessaries prescribed for vaginal thrush/candidiasis, for the all leukaemia category. This was not explained by maternal age, a possible indicator of increased levels of hospital care and, consequently, increased frequency of recording. The presence of candida may act as a marker for other vaginal infections (Cotch et al, 1998) and increase exposure to infections in vaginally delivered babies. Adjustment for type of delivery did not alter the OR for antifungal medication, which provides limited support for the theory that exposure to infectious agents reduces the risk of childhood ALL.

Few other studies have reported on neonatal or early infections. Exposure to specific viral infections under 6 months of age, as reported at interview, has been noted to increase the risk of leukaemia/lymphoma (McKinney et al, 1987). This is in contrast to a Dutch study from a postal questionnaire showing that children with leukaemia experienced fewer infections in the first year of life (van Steensel-Moll et al, 1986).

Reports of increased risk of leukaemia for first-born children have been inconsistent (Fasal et al, 1971; Shaw et al, 1984; Roman et al, 1997; Westergaard et al, 1997). Our results for neither first pregnancies nor first live births were statistically significant for leukaemia (or ALL). Children born first in a family are likely to have lower exposure to infections than those of a higher birth order and our findings of lowered risk for not being first born are weakly supportive of the delayed infectious exposure hypothesis.

The following observations summarize the evidence consistent with the prior hypothesis of a low frequency of infections increasing risk. Significantly reduced ORs for neonatal infections, particularly in the 0 to 4-year-olds, and the lowered risk for anti-fungal use in pregnancy are the strongest signals of support, with parity providing weaker corroboration.

No association for all leukaemias or ALL was found with high birthweight, in common with some (McKinney et al, 1987; Golding et al, 1990; Zack et al, 1991; Roman et al, 1997), but not all studies (Fasal et al, 1971; Daling et al, 1984; Shu et al, 1988; Kaye et al, 1991; Cnattingius et al, 1995; Ross et al, 1996; Westergaard et al, 1997), although high birthweight has been linked to acute myeloid leukaemia (Roman et al, 1997; Westergaard et al, 1997) and children diagnosed with cancers under 2 years (Yeazel et al, 1997). Ross and colleagues (Ross et al, 1996) argue for a biological mechanism involving insulin-like growth factor (IGF-1) to explain risks correlated with high birthweight.

Perinatal hypoxia was not associated with childhood leukaemia but was linked to ‘other and unspecified solid tumours’ in a Swedish birth registry study (Forsberg and Kallen, 1990). This contrasts with the present results where both poor Apgar scores and birth asphyxia recorded in the notes raised the risk for leukaemias. The reason for these associations is not clear and it is notable the effect did not persist to the Apgars at 5 min.

Childhood leukaemia and maternal use of drugs in pregnancy and during labour have been extensively studied with links observed for the following labour drugs – inhaled nitrous oxide anaesthesia (Zack et al, 1991), narcotic analgesia (McKinney et al, 1987; Gilman et al, 1989; Golding et al, 1990) and sedatives and tranquillizers in pregnancy (McWhirter and Chant, 1990). The present study lent little support to the concept of transplacental leukaemogenesis effected by maternal drug use.

### Lymphomas

The finding that significantly more mothers of control children received ultrasound scans is not supported by other published literature (McKinney et al, 1987; Shu et al, 1994). The majority of examinations were conducted in the second trimester and increasing numbers of scans conferred greater protection. The explanation for this is unlikely to be biological and ultrasound examinations may well be markers of other exposures. The separate ORs for Hodgkin's disease and non-Hodgkin's lymphoma are both less than unity, but neither are statistically significant. A recent study of non-Hodgkin's lymphoma revealed few maternal or perinatal risk factors (Adami et al, 1996).

### Central nervous system tumours

The only maternal or neonatal factor associated with risk of developing a CNS tumour was an inverse association with use of antacids during labour. The absence of any other significant risks is consistent with the findings of McCredie et al (1994*a*, 1994*b*). No association was found for anti-nausea medication during pregnancy, thus failing to support the findings from an American study of children with astrocytomas (Kuijten et al, 1990). N-nitrosatable drugs have been postulated as transplacental carcinogens, but neither this or another study (Carozza et al, 1995) found an elevated risk. Use of narcotic analgesics during pregnancy and labour have been linked specifically to childhood CNS tumours (Linet et al, 1996), but our study results were non-significant. Neonatal infections and neonatal distress observed as increasing risk by Linet et al (1996) had non-significant ORs close to unity in the current study.

### Other solid tumours

The analysis of a solid tumour group was pragmatic, as, although subtypes within this subgroup may have differing aetiologies, the consequence of further subdivision would have been unacceptably low power. Some infections in pregnancy are known to have teratogenic consequences; for example, rubella and cytomegalovirus, but the search for in-utero infections which may be transplacental carcinogens has been unsuccessful. The current study has shown a significantly raised risk of infections in pregnancy for the other solid tumours, accounted for by the respiratory tract. Interpretation is difficult as the confidence intervals are wide, particularly for the respiratory tract infections, and the infections relate to a mixed group of tumour types, none of which predominated, indicating a causal link is improbable.

Neonatal skin infections were associated with a significantly increased risk for the other solid tumours. This was unsupported by other results in this exposure category as the finding was not restricted to any particular tumour type and the OR for 'any neonatal infection' was in the opposite direction.

### CONCLUSIONS

Overall, few specific maternal or neonatal risks were statistically significant for individual disease subgroups. This may represent a real effect implying that early exposures are not important determinants of risk or simply reflect a lack of statistical power. The possibility remains of there being risks associated with factors which either are unmeasured or were represented by insufficient numbers in the current study; for example, maternal ingestion of a specific drug. The most notable finding was a negative association with neonatal infections and leukaemia and ALL. This supports the concept of infectious exposure in early life conferring protection against these malignancies. The combination of data from this and other similar population-based studies will improve power and, crucially, improved size will permit examination by diagnostic groups that are more clearly defined both at the histological and molecular level.

### ACKNOWLEDGEMENTS

We are grateful to all the individuals who have assisted with our study and who are too numerous to be named separately. The support and collaboration of the clinicians from the UK Children's Cancer Study Group (UKCCSG) has been invaluable. Interviewers and all study staff at the Information and Statistics Division of the National Health Service in Scotland especially Bea Jardine and Nadine Hunter are thanked in addition to the Cancer Surveillance Group and the Directors of the Regional Cancer Registries and their staff. Consultants, GPs and medical staff, in particular medical records staff, in hospitals throughout Scotland have provided extensive assistance. Eve Roman is thanked for the use of the data collection instrument and helpful discussion. Roger Black and David Brewster kindly commented on previous drafts of the paper. The study is a participating centre in the United Kingdom Childhood Cancer Study (UKCCS) which is sponsored and administered by the United Kingdom Co-ordinating Committee on Cancer Research. All participating families are thanked for making this research possible. Financial support was received from The Scottish Office Department of Health (principal funders), The Chief Scientist Office (SODH), Scottish Power plc, Scottish Nuclear Limited and Scottish Hydro-Electric plc.

#### REFERENCES

Adami J, Glimelius B, Cnattingius S, Ekbom A, Zahm SH, Linet M and Zack M (1996) Maternal and perinatal factors associated with non-Hodgkin's lymphoma among children. *Int J Cancer* **65**: 774–777

Birch JM and Marsden HB (1987) A classification scheme for childhood cancer. *Int J Cancer* **40**: 620–624

Breslow NE and Day NE (1980) *Statistical Methods in Cancer Research, Vol. 1. The Analysis of Case-control Studies*. *IARC Scientific Publications*, No 32. IARC: Lyon

*British National Formulary Number 24* (1992) British Medical Association and the Royal Pharmaceutical Society of Great Britain

Carozza SE, Olshan AF, Faustman EM, Gula MJ, Kolonel LN, Austin DF, West ED, Weiss NS, Swanson GM, Lyon JL, Hedley-Whyte T, Gilles FH, Aschenbrener C and Leviton A (1995) Maternal exposure to N-nitrosatable drugs as a risk factor for childhood brain tumours. *Int J Epidemiol* **24**: 308–312

Carstairs V and Morris R (1991) *Deprivation and Health in Scotland*. Aberdeen University Press: Aberdeen

Cnattingius S, Zack MM, Ekbom A, Gunnarskog J, Krewger A, Linet M and Adami H-O (1995) Prenatal and neonatal risk factors for childhood lymphatic leukaemia. *J Natl Cancer Inst* **87**: 908–914

Cotch MF, Hillier SL, Gibbs RS and Eschenbach DA (1998) Epidemiology and outcomes associated with moderate to heavy Candida colonisation during pregnancy. Vaginal infections and prematurity study group. *Am J Obstet Gynecol* **178**: 374–380

Daling JR, Starzyk P, Olshan AF and Weiss NS (1984) Birthweight and the incidence of childhood cancer. *J Natl Cancer Inst* **72**: 1039–1041

*© 1999 Cancer Research Campaign*

Doll R (1989) The epidemiology of childhood leukaemia. *J R Statist Soc A Part* **3**: 341–351

Fasal E, Jackson EW and Klauber MR (1971) Birth characteristics and leukaemia in childhood. *J Natl Cancer Inst* **47**: 501–509

Forsberg J-G and Kallen B (1990) Pregnancy and delivery characteristics of women whose infants develop cancer. *APMIS* **98**: 37–42

Gilman EA, Kinnier-Wilson LM, Kneale GW and Waterhouse JAH (1989) Childhood cancers and their association with pregnancy drugs and illnesses. *Paediatr Perinatal Epidemiol* **3**: 66–94

Golding J, Paterson M and Kinlen L (1990) Factors associated with childhood cancer in a national cohort study. *Br J Cancer* **62**: 304–308

Greaves MF and Alexander FE (1993) An infectious aetiology for common acute lymphoblastic leukaemia in childhood? *Leukaemia* **7**: 349–360

Greaves MF (1997) Aetiology of acute leukaemia. *Lancet* **349**: 344–349

WHO (1994) *International Statistical Classification of Diseases and Health-Related Problems*. World Health Organization: Geneva

Kaye SA, Robison L, Smithson NA, Gunderson P, King FL and Neglia JP (1991) Maternal reproductive history and birth characteristics in childhood acute lymphoblastic leukaemia. *Cancer* **68**: 1351–1355

Kuijten RR, Bunin GR, Nass CC and Meadows AT (1990) Gestational and familial risk factors for childhood astrocytoma: results of a case control study. *Cancer Res* **50**: 2608–2612

Linet MS, Gridley G, Cnattingus S, Nicholson HS, Martinsson U, Glimelius B, Adami H-O and Zack M (1996) Maternal and perinatal risk factors for childhood brain tumours (Sweden). *Cancer Causes Control* **7**: 437–448

McCredie M, Maisonneuve P and Boyle P (1994*a*) Perinatal and early postnatal risk factors for malignant brain tumours in New South Wales children. *Int J Cancer* **56**: 11–15

McCredie M, Maisonneuve P and Boyle P (1994*b*) Antenatal risk factors for malignant brain tumours in New South Wales children. *Int J Cancer* **56**: 6–10

McKinney PA, Cartwright RA, Saiu J, Mann JR, Stiller CA, Draper GJ, Hartley AL, Hopton PA, Birch JM, Waterhouse JAH and Johnston HE (1987) The inter-regional epidemiological study of childhood cancer (IRESCC): a case control study of aetiological factors in leukaemia and lymphoma. *Arch Dis Childhood* **62**: 279–287

McKinney PA, Smith K and Findlay E (1995) The Scottish case control study of childhood leukaemia and cancer: methodology and environmental measures of exposure. *Health Bull* (Scotland) **53**: 222–229

McKinney PA, Juszczak E, Findlay E and Smith K (1998) Case-control study of childhood leukaemia and cancer in Scotland: findings for neonatal intramuscular vitamin K. *Br Med J* **316**: 173–177

McWhirter WR and Chant DC (1990) A study of antental factors associated with the occurrence of childhood leukaemia. *Cancer Therapy Control* **1**: 133–139

Roberts HR, Rushton L, Muir KR, Dengler R, Coupland CAC, Jenkinson CM, Ruffell A and Chilvers CED (1995) The use of family health services authority registers as a sampling frame in the UK: a review of theory and practice. *J Epid Com Health* **49**: 344–347.

Roman E, Ansell P and Bull D (1997) Leukaemia and non-Hodgkin's lymphoma in children and young adults: are prenatal and neonatal factors important determinants of disease. *Br J Cancer* **76**: 406–415

Ross JA, Perentesis JP, Robison LL and Davies SM (1996) Big babies and infant leukaemia; a role for insulin-like growth factor-1. *Cancer Causes Control* **7**: 553–559

Shaw G, Lavey R, Jackson R and Austin D (1984) Association of childhood leukaemia with maternal age, birth order and paternal occupation. *Am J Epidemiol* **119**: 788–795.

Shu XO, Gao YT, Brinton LA, Linet MS, Tu JT, Zheng W and Fraumeni Jr JF (1988) A population-based case control study of childhood leukaemia in Shanghai. *Cancer* **62**: 635–644.

Shu X-O, Jun F, Linet MS, Zheng W, Clemens J, Mills J and Gao Y-T (1994) Diagnostic x-ray and ultrasound exposure and risk of childhood cancer. *Br J Cancer* **70**: 531–536.

Stata Corp. (1997) Stata Statistical Software: release 5.0. Stata Corporation College Station, TX.

Stiller CA, Allen MB and Ebtock EM (1995) Childhood cancer in Britain: the National Registry of Childhood Tumours and incidence rates 1978–1987. *Eur J Cancer* **202**: 8–34.

Van Steensel-Moll HA, Valkenburg HA and van Zanen GE (1986) Childhood leukaemia and infectious diseases in the first year of life: a register based case control study. *Am J Epidemiol* **124**: 590–594.

Westergaard T, Anderson PK, Pederson JB, Olsen JH, Frisch M, Sorenson HT, Wohlfahrt J and Melbye H (1997) Birth characteristics, sibling patterns and acute leukaemia risk in childhood: a population-based cohort study. *J Natl Cancer Inst* **89**: 939–947.

Yeazel MW, Ross JA, Buckley JD, Woods WG, Ruccione K and Robison LL (1997) High birthweight and risk of specific childhood cancers: a report from the Children's cancer study group. *J Pediatr* **131**: 671–677

Zack M, Adami HO and Ericson A (1991) Maternal and perinatal risk factors for childhood leukaemia. *Cancer Res* **51**: 3696–3701

*© 1999 Cancer Research Campaign*
